# Do Multidisciplinary Team (MDT) processes influence survival in patients with colorectal cancer? A population-based experience

**DOI:** 10.1186/s12885-015-1683-1

**Published:** 2015-10-13

**Authors:** Alastair Munro, Mhari Brown, Paddy Niblock, Robert Steele, Frank Carey

**Affiliations:** Tayside Cancer Centre, Ninewells Hospital and Medical School, Dundee, DD1 9SY UK; University of Dundee Ninewells Hospital and Medical School, Dundee, DD1 9SY UK; University of Dundee Ninewells Hospital and Medical School, Mailbox 4 Level 7, Dundee, DD1 9SY UK; Ninewells Hospital and Medical School, Dundee, DD1 9SY UK

## Abstract

**Background:**

MDT (multidisciplinary team) meetings are considered an essential component of care for patients with cancer. However there is remarkably little direct evidence that such meetings improve outcomes. We assessed whether or not MDT (multidisciplinary team) processes influenced survival in a cohort of patients with colorectal cancer.

**Methods:**

Observational study of a population-based cohort of 586 consecutive patients with colorectal cancer diagnosed in Tayside (Scotland) during 2006 and 2007.

**Results:**

Recommendations from MDT meetings were implemented in 411/586 (70.1 %) of patients, the MDT+ group. The remaining175/586 (29.9 %) were either never discussed at an MDT, or recommendations were not implemented, MDT- group. The 5-year cause-specific survival (CSS) rates were 63.1 % (MDT+) and 48.2 % (MDT-), *p* < 0.0001. In analysis confined to patients who survived >6 weeks after diagnosis, the rates were 63.2 % (MDT+) and 57.7 % (MDT-), *p* = 0.064. The adjusted hazard rate (HR) for death from colorectal cancer was 0.73 (0.53 to 1.00, *p* = 0.047) in the MDT+ group compared to the MDT- group, in patients surviving >6 weeks the adjusted HR was 1.00 (0.70 to 1.42, *p* = 0.987). Any benefit from the MDT process was largely confined to patients with advanced disease: adjusted HR _(early)_ 1.32 (0.69 to 2.49, *p* = 0.401); adjusted HR_(advanced)_ 0.65 (0.45 to 0.96, *p* = 0.031).

**Conclusions:**

Adequate MDT processes are associated with improved survival for patients with colorectal cancer. However, some of this effect may be more apparent than real – simply reflecting selection bias. The MDT process predominantly benefits the 40 % of patients who present with advanced disease and conveys little demonstrable advantage to patients with early tumours. These results call into question the current belief that all new patients with colorectal cancer should be discussed at an MDT meeting.

## Background

The introduction of routine Multidisciplinary Team (MDT) meetings into cancer care in the UK followed the publication of the Calman-Hine Report [1]. The assumption was that regular MDT meetings, at which all new patients with cancer would be discussed, would be an effective method of extending the benefits of “specialist care” [2] (however that might be defined) to all patients with cancer. By 2000, the National Cancer Plan [3] contained the instruction: “from 2001 put in place site-specific multidisciplinary teams and ensure all patients are reviewed by them”. The assumption was that the MDT process would improve survival rates for patient with cancer in the UK.

There are now over 200 publications assessing, or claiming to assess, the benefits associated with MDT meetings (“tumor boards” in the USA) for patients with cancer. These papers range across a wide variety of tumour types, however only six papers [4–9] describe the effect of MDT discussion upon survival in patients with colorectal cancer. Their main features are summarised in Table 1. Given the relative paucity of available evidence, we have reviewed the effect of MDT discussion, and implementation of recommendations, on survival in a population-based cohort of patients with colorectal cancer who were diagnosed in 2006 and 2007, and for whom we have data from long-term follow-up.

**Table 1:** Details of studies on the relationship between MDT discussion and outcome in patients with colorectal cancer

Author	Country	Setting	Period	Patients	Comparison	Factors significant in MVA	Survival outcome	HR death any cause (95 % c.i.)
Ye	China	Hospital-based	1999–2006	after radical resection for colorectal cancer	before MDT introduced in 2002 (*n* = 297) cf. after MDT (*n* = 298)	MDT, Age, Differentiation, Number of nodes examined, Stage	OS	0.62 (0.46 to 1.48)
Du	China	Hospital-based	2001–2005	with resectable locally advanced rectal cancer	contemporaneous patients; *n* = 101 were evaluated by MDT members and were treated with neoadjuvant chemotherapy; *n* = 162 were not evaluated	EMVI, pre-treatment CEA, pathological TNM stage	OS, DFS	0.88 (0.52 to 1.48)
Lordan	England	Hospital-based	1996–2006	with hepatic metastases from colorectal cancer who were referred for liver surgery	those who were referred by a team which contained a HPB surgeon (*n* = 108); those who were referred by teams lacking a HPB surgeon (*n* = 223)	recurrence, septicaemia, pre-operative chemotherapy, referral via team with HPB surgeon, macroscopic invasion of diaphragm	OS, DFS	0.85 (0.60 to 1.19)
McDermid	Scotland	Surgeon-based	1997–2005	with resected colorectal cancers (excluding Dukes’A)	before MDT introduced in 2002 (*n* = 176) cf. after MDT (*n* = 134)	Age, stage, MDT	OS	0.73 (0.54 to 0.99)
Palmer	Sweden	Regional	1995–2004	with rectal cancer invading into adjacent organs	3 groups 1) *n* = 65 discussed at MDT appropriately staged 2) *n* = 99 appropriately staged not discussed at MDT 3) *n* = 139 not appropriately staged (whether or not discussed at MDT)	Age	OS (CSS for MVA)	0.95 (0.62 to 1.45)
Wille-Jorgensen	Denmark	Hospital	2001–2006	Rectal cancer	Before MDT introduced (*n* = 467) c.f.after MDT introduced (*n* = 344)	No MVA	OS	0.94 (0.79 to 1.12)

*OS* Overall Survival, *DFS* Disease-free Survival, *CSS* Cause-specific survival, *MVA* Multivariate Analysis, *EMVI* Extramural vascular invasion, *HPB* Hepatobiliary, *CEA* Carcinoembryonic antigen, *HR* Hazard ratio (event is death and comparator is no MDT discussion)

## Methods

We performed a retrospective review of prospectively acquired data. Tayside is a geographically defined region of Eastern Scotland. Since 1997 there has been a weekly colorectal MDT meeting at which all newly diagnosed patients within the region are discussed. All pathology, including that from the very few private patients, is discussed at the MDT. The information we have gathered reflects a regional, population-based, experience.

All patients in Tayside have a unique identification number (the CHI number) which can be used to link individual patient’s records across multiple databases. We used hospital information systems to obtain information on all patients with a diagnosis of colorectal cancer in Tayside between 1st January 2006 and 31st December 2007 this approach has been approved by the Caldicott Guardian and the Tayside Regional Ethics Committee (REC reference 06/S1402/3). This project was classified as clinical audit and therefore written informed consent from patients was not required. The data analysed in this study are not publicly available.

We obtained data on MDT discussions and recommendations from worksheets filled in by senior clinicians (PN, AM) at each MDT meeting. We accepted the following as definitions of “recommendation”: surgery; radiotherapy; chemotherapy; neoadjuvant therapy; for oncological opinion; for further investigation; palliative care; follow-up only. Each patient, rather than each discussion, was the unit of analysis.

We staged patients using the TNM system (5th edition) [10] from which we generated Dukes’ stage; we scored co-morbidity using the ACE-27 system [11]. Linkage via postcode provided information on income deprivation using Scottish Index of Multiple Deprivation (SIMD) data from 2006 [12]. Using hospital notes, radiology information systems, oncology electronic patient records, and other hospital-based documentation, we assessed whether or not the initial recommendation made by the MDT had, or had not, been implemented. Data on outcomes came from the electronic patient records and central hospital information systems. We entered the anonymised data into a FileMaker Pro database (FileMaker Inc.) and exported the data to Stata (StataCorp) for statistical analyses.

Statistical analyses included: Fisher’s exact test for tabular comparisons; Mann–Whitney test for comparison of group means; the Kaplan-Meier method for constructing survival curves; the logrank test for comparison of survival curves; Cox’s proportional hazards model for multivariate analysis of prognostic factors.

## Results

We identified 586 patients (311 males; 275 females) newly diagnosed with colorectal cancer between 1st January 2006 and 31st December 2007: 337 patients have died; 230 from colorectal cancer and 107 from other causes. The surviving patients have been followed up for a median of 74 months (range 16 to 91; mean 73.3). Of the 586 patients, 513 were discussed at an MDT meeting. For the majority of patients discussed at MDT meetings there was clear evidence of a definite recommendation being made: for only 31/513 (6.0 %) of discussions was it impossible to identify a recommendation. The MDT recommendation was implemented in 411/586 (70.1 %) of patients; the recommendation was not implemented in 102/586 (17.4 %) of patients and 73/586 (12.5 %) of patients were never discussed at an MDT. For simplicity of analysis, we have merged the group who were never discussed with the group who were discussed, but in whom the MDT recommendation was not implemented: the MDT process could contribute little, if anything, to outcomes for these patients.

Table 2 summarises the demographic and clinical characteristics of the patients according to two groups: MDT+ (discussed at an MDT with evidence of an implemented recommendation); MDT- (either not discussed or no implemented recommendation). The groups differed significantly in age, stage, histological grade and comorbidity. There were no significant differences in gender, tumour site, or income deprivation.

**Table 2:** Clinico-pathological variables according to group – *p* values are from Fisher’s exact test and Mann–Whitney test. Staging is according to the Dukes’ system

	Total		MDT+		MDT-		*P* value
Mean age	70.6	(69.6 to 71.6)	68.6	(67.4 to 69.8)	75.3	(73.7 to 76.9)	<0.0001
Gender	N		N		N		
Female	275	46.9 %	191	46.5 %	84	48.0 %	0.786
Male	311	53.1 %	220	53.5 %	91	52.0 %	
Stage							
Early or A or B	213	36.3 %	148	36.0 %	65	37.1 %	<0.0001
C	135	23.0 %	112	27.3 %	23	13.1 %	
Neoadjuvant	38	6.5 %	35	8.5 %	3	1.7 %	
Advanced or metastatic	200	34.1 %	116	28.2 %	84	48.0 %	
Grade							
Well or moderate	427	72.9 %	320	77.9 %	107	61.1 %	<0.0001
Poor	87	14.9 %	65	15.8 %	22	12.6 %	
Unknown	49	8.4 %	23	5.6 %	26	14.9 %	
No histology	23	3.9 %	3	0.7 %	20	11.4 %	
Site							
Right colon	200	34.1 %	137	33.3 %	63	36.0 %	0.785
Left colon	196	33.5 %	140	34.1 %	56	32.0 %	
Rectum	174	29.7 %	124	30.2 %	50	28.6 %	
Unspecified	16	2.7 %	10	2.4 %	6	3.4 %	
Income deprivation quintile							
Least deprived	114	19.5 %	79	19.2 %	35	20.0 %	0.889
2nd	151	25.8 %	107	26.0 %	44	25.1 %	
3rd	123	21.0 %	91	22.1 %	32	18.3 %	
4th	94	16.0 %	64	15.6 %	30	17.1 %	
Most deprived	81	13.8 %	55	13.4 %	26	14.9 %	
Unknown	23	3.9 %	15	3.7 %	8	4.6 %	
ACE-27 comorbidity score							
0	162	27.7 %	132	32.1 %	30	17.1 %	<0.0001
1	201	34.3 %	151	36.7 %	50	28.6 %	
2	93	15.9 %	59	14.4 %	34	19.4 %	
3	53	9.0 %	31	7.5 %	22	12.6 %	
Unknown	77	13.1 %	38	9.3 %	39	22.3 %	
Total	586	100.0 %	411	70.1 %	175	29.9 %	

**Fig. 1:**
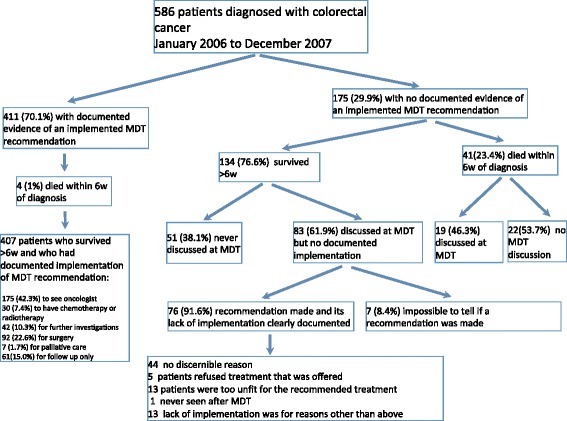
Flow chart illustrating the population of patients and outcomes

Figure 1 illustrates the routes followed by patients following initial diagnosis. We partitioned the analysis according to whether or not patients survived more than 6 weeks after the date of diagnosis. This is because patients who die soon after diagnosis may not be discussed in the MDT and this could artefactually lower survival rates in the group of patients defined as “not discussed”. Forty five patients died within six weeks of diagnosis: their mean age was 77.7 years (range 55 to 91; median 79); 22 were female and 23 were male; 31 had advanced or metastatic disease, 14 had early disease; 28 died from rapidly progressive disease, 11 died from complications following surgery and 6 died from co-morbid conditions. Only 4 of these 45 patients (8.9 %) were in the MDT+ group, 41/45 (91.1 %) were in the MDT- group.

Overall survival at 5 years was significantly better in the MDT+ group, 52.2 % (95 % confidence interval 47.3 to 56.7 %), than in the MDT- group, 33.6 % (95 % confidence interval 26.7 to 40.6 %); logrank *p* value <0.00001. The hazard ratio adjusted for age, gender, stage, tumour site, grade, socioeconomic deprivation and co-morbidity was 0.72 (95 % confidence interval 0.56 to 0.92; *p* = 0.009) for the MDT+ group compared with the MDT- group.

**Fig. 2:**
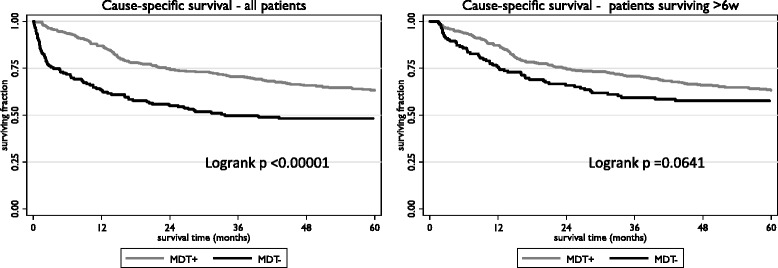
Kaplan-Meier survival curves for cause-specific survival according to MDT group

The 5-year cause-specific survival (CSS) for the MDT+ group was 63.1 % (95 % confidence interval 58.0 to 67.8 %); for the MDT- group the rate was 48.2 % (95 % confidence interval 40.2 to 55.8 %). This difference was statistically significant: *p* value by logrank test < 0.00001. The corresponding survival curves are shown in Fig. 2. In analysis confined to patients surviving for more than 6 weeks after diagnosis the cause-specific survival rates at 5 years are: MDT+ group 63.2 % (95 % confidence interval 58.1 to 67.9 %); for the MDT- group the rate was 57.7 % (95 % confidence interval 48.6 to 65.7 %). This difference was not statistically significant: *p* value by logrank test = 0.064.

In univariate analysis the hazard ratio (HR) for death from colorectal cancer was 0.53 (95 % confidence interval 0.40 to 0.69; *p* < 0.0001) when patients in the MDT+ group were compared with those in the MDT- group. This effect was attenuated by adjustment (for age, gender, tumour stage, tumour site, tumour grade, income deprivation and comorbidity) in multivariate analysis: HR 0.73 (95 % confidence interval 0.53 to 1.00; *p* = 0.047). The corresponding figures, considering only those patients who survived for more than 6 weeks, were: univariate HR 0.75 (95 % confidence interval 0.55 to 1.01; *p* = 0.065); adjusted HR 1.00 (95 % confidence interval 0.70 to 1.42; *p* = 0.987).

**Fig. 3:**
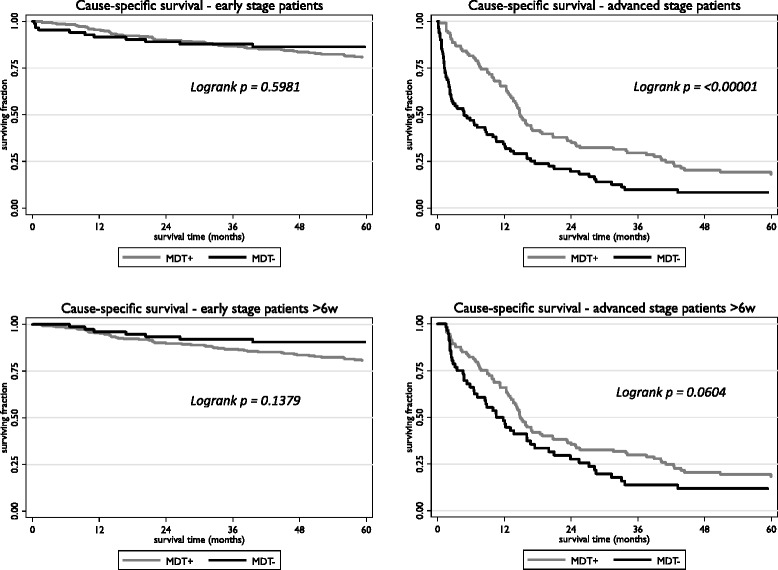
Kaplan-Meier cause-specific survival curves according to extent of disease (for definition see text) and MDT group

We further divided the patients, according to stage at presentation into two main groups: 386 patients with operable or early disease (T_1-4_N_x,0-2_M0) – the “early” group; 200 patients with locally advanced (inoperable) or metastatic disease (T_unknown_N_unknown_M0; T_any_N_any_M1) – the “advanced” group. The 5 year cause-specific survival for the patients with advanced disease was 14.3 %; for the patients with early disease it was 81.8 %. Figure 3 shows the cause-specific survival curves for early and advanced patients. The survival rates and adjusted hazard ratios are summarised in Table 3.

**Table 3:** Five year Cause-specific survival (CSS) rates with *p* values from logrank test. The hazard ratios (HR) their 95 % confidence intervals and associated *p* values were estimated using the Cox proportional hazards model with adjustment for age, gender, grade and site of tumour, income deprivation, co-morbidity and, where appropriate, stage. The MDT+ group is compared to the MDT- group and so a hazard rate <1.00 indicates survival benefit from MDT discussion and implementation

		5 year CSS		Logrank p	Adjusted HR	*p* value
All patients		MDT+	MDT-			
	All stages	63.1 %	48.2 %	<0.0001	0.73 (0.53 to 1.00)	0.047
	Early	80.6 %	86.4 %	0.598	1.32 (0.69 to 2.49)	0.401
	Advanced	18.0 %	8.4 %	<0.00001	0.65 (0.45 to 0.96)	0.031
Survived >6w						
	All stages	63.2 %	57.7 %	0.064	1.00 (0.70 to 1.42)	0.987
	Early	80.6 %	90.6 %	0.138	1.85 (0.88 to 3.88)	0.105
	Advanced	18.2 %	11.8 %	0.064	0.89 (0.58 to 1.36)	0.590

Only three patients were apparently denied the opportunity for adjuvant treatment because they were not discussed at an MDT. One is alive with no evidence of disease at 6.5 years, one died of an unrelated cause 2.8 years after diagnosis, and one patient died from rectal cancer 11 months after diagnosis. Despite the lack of MDT discussion, this patient was reviewed post-operatively in the oncology department. When seen and assessed the patient had diarrhoea and weight loss following a colo-anal anastomosis: the patient was not considered sufficiently fit for adjuvant chemotherapy, her disease later recurred and she was treated with palliative radiotherapy.

## Discussion

### Main points

We present here a population-based approach to the question of whether or not colorectal MDT meetings improve survival in patients with colorectal cancer. These results reflect those achieved by a mature MDT, a group of clinicians who had been working together for over eight years. We have included comorbidity and socio-economic deprivation as well as other more widely reported demographic and clinico-pathological variables in our analysis. We found no evidence that patients with potentially curable tumours suffer harm as a result of failure in the MDT process. There is some apparent benefit from MDT discussion in patients with advanced or metastatic disease, but the evidence is insufficient to determine whether this is an artefact arising from selection bias or whether the advantage is genuine. The statistical treatment of deaths within six weeks of diagnosis had an important effect on the estimate of the magnitude of the effect associated with the MDT process. Censoring deaths occurring within six weeks of diagnosis attenuated the estimated benefit: mainly because only a small proportion (<10 %) of patients who died within 6 weeks had evidence of an implemented MDT recommendation. This is important for two reasons. Firstly, it is unlikely that the MDT process could, *per se*, have had any influence over the occurrence of these early deaths. Secondly, including these patients, whose fates were largely sealed by the time of diagnosis, introduces a selection bias which will exaggerate the perceived benefits associated with the MDT process.

### Limitations

This is an observational study and, as such, is subject to bias. The group of patients (MDT-) who were not discussed at an MDT meeting, or where MDT recommendations were not implemented, was not derived through random allocation, and it is highly likely that membership of this group was influenced by confounding variables not considered in the analysis. Although nearly 600 patients are included in this study, there are only 175 patients in the MDT- group and so statistical comparisons may be relatively underpowered.

We adopted a loose definition of what constituted a “recommendation”. We did not stipulate that the MDT had to define a specific plan for treatment and accepted that, bearing in mind that patients themselves may not have been adequately represented at the MDT [13], it is reasonable to consider that further discussion with an oncologist could constitute an outcome.

By using cause-specific survival as the outcome of interest for the study, we excluded consideration of whether MDT discussion might have had an impact on the morbidity of treatment by, for example, recommending rectum-conserving surgery rather than abdomino-perineal excision. However, the prime purpose behind the introduction of MDT meetings was to improve survival and this is therefore the standard by which the process should be judged.

These patients were assessed and managed in the pre-biological era of treatment for colorectal cancer. All patients had access to standard chemotherapeutic agents and to conformally-planned radiotherapy. However, at the time of initial decision-making, biological agents (such as cetuximab and bevacizumab) had not been approved for use in Scotland. Pathological specimens were not routinely assessed for molecular markers – primarily because no targeted agents were available for treatment.

### Strengths

This is a population-based study and the results and conclusions may therefore have more general relevance than those from studies based on data from a single hospital. We have complete follow-up, including details of vital status, for all patients. We have been able to document not just whether the patient was discussed at an MDT meeting, or whether a recommendation was made, but also whether or not any recommendation was implemented. The analysis covers a short time period, only two years, and all patients were cared for by the same team of oncologists and surgeons. There is therefore consistency of decision-making and clinical management.

### General discussion

There is a dramatic difference in long-term survival when the experience of patients with early disease is compared with that of patients who present with advanced or metastatic disease: the 5 year survival for the 200 patients with advanced disease was 14.3 %, the corresponding figure for the 386 patients with early disease was 81.8 %. The magnitude of this difference may go some way to explaining the observed differences in colorectal survival when comparisons are made between institutions, or amongst nations [14]. Any underreporting or exclusion of patients with advanced or metastatic disease will significantly inflate the overall estimates of survival after a diagnosis of colorectal cancer.

We have presented results for all patients, and for that subset of patients who survived for at least six weeks after diagnosis. By excluding patients who died within six weeks of diagnosis we eliminate from consideration patients who presented as emergencies and who died within a few weeks of surgery, as well as those patients who presented in the terminal phase of their illness. It is unreasonable to expect that MDT discussion would improve outcomes for such patients, their fates are determined by events that are usually beyond the control of any MDT.

When survival analysis was restricted to patients who survived for at least six weeks after diagnosis any benefits associated with MDT discussion were less evident. This suggests that, in a population-based series such as this one, MDT discussion is to some extent an indicator of longer-term survival. Patients who die at and around the time of diagnosis are less likely to be discussed at an MDT meeting. This is consistent with the finding that MDT discussion and implementation of recommendations was less likely in patients with higher levels of comorbidity (Table 2).

Our results are consistent with the published literature [4–9] in patients with colorectal cancer: the MDT process is associated with improved survival. However, we clearly demonstrate that in patients with non-metastatic disease the MDT process contributes little to cancer-specific survival (Fig. 3 and Table 3). The apparent benefit of the MDT discussion is most marked in patients with advanced disease. This benefit is still apparent, although not statistically significant, when those patients who die within six weeks of diagnosis are excluded from analysis. This suggests that the main contribution of the MDT may be to co-ordinate the management of patients with complex clinical problems – potentially resectable liver metastases, tumours of borderline operability. Clinicians are not always aware of what their colleagues in other specialties might have to offer [15]. A recent Australian study [16] looking at the effect of MDT discussion upon management decisions drew similar conclusions. MDT discussions were of more value for patients with more complex clinical problems. Of course it is entirely possible that the observation of benefit in this group is due to hidden confounding – only those patients who, on *a priori* grounds, are expected to benefit are discussed, the others are not. The “beneficial effect” of discussion might simply be a self-fulfilling prophecy.

Two papers [17, 18] have looked at implementation rates for MDT recommendations. In the study from Plymouth [17] the implementation rate was 44/47 (93.6 %), the rate in the study from Bristol [18] was 137/157 (87.3 %). The rate in our study, confining analysis to patients surviving for at least six weeks, was 407/490 (83.1 %).

Our results suggest that much of the workload of MDT meetings, as they are currently constituted, may have little impact upon cancer-specific survival in patients with colorectal cancer. For the group of 386 patients with early disease, 66 % of all patients discussed, the presence or absence of adequate MDT process had no significant effect on survival. MDT discussion of all new patients is an instrument of cultural change and has helped to establish an environment in which equality of access to a uniform standard of care is now considered the norm. There may be more cost-effective ways to maintain this new culture. Coordination of care is important [19] but does not necessarily require the full majesty of an MDT meeting. Decision-support systems [20–22] could easily be used outwith a formal MDT meeting. For more complex problems, an asynchronous virtual MDT [23] might offer a more flexible and less labour-intensive approach than weekly face-to-face meetings.

Population-based screening may bring with it a new set of problems – primarily related to pathological interpretation of early disease [24]. In the future there may be an increase in the number of patients with early disease who present complex problems requiring MDT discussion [25]. This indicates the need for a flexible approach to the role, remit and constitution of the MDT. It will not be easy to modify the role of the traditional MDT. Assumptions concerning the value of this practice are now firmly embedded in the procedures for assessing the quality of cancer services: the Scottish Quality Performance Indicator (QPI) sets a target of 95 % of new patients with colorectal cancer being discussed at an MDT [26]; the colorectal MDTs in England are assessed against a set of 43 measures, all of which concern process rather than outcome [27].

## Conclusions

In summary, we conclude that: the MDT process is associated with, but not necessarily the cause of, improved survival in patients with advanced or metastatic colorectal cancer; neither discussion at an MDT, nor evidence for an implemented recommendation, significantly affects survival in the 66 % of patients who present with early or localised disease; much of the time currently spent in MDT discussion may be futile, a more focussed approach to discussion might represent better value for money; in common with others [16, 17] we believe that we should now reconsider the value of routine discussion in an MDT meeting of all newly diagnosed patients with colorectal cancer.

## Abbreviations

MDT: Multidisciplinary team
CSS: Cause-specific survival – in which death from colorectal cancer is coded as an event
HR: Hazard ratio
OS: Overall survival
DFS: Disease-free survival
MVA: Multivariate analysis
EMVI: Extramural vascular invasion
HPB: Hepatobiliary
CEA: Carcinoembryonic antigen
